# A Rare Case of a Massive Food Bolus Mimicking Lung Cancer

**DOI:** 10.7759/cureus.11043

**Published:** 2020-10-19

**Authors:** Abdul Karim Arida, Omar Khaddam, Sarah Al Naher, Ashraf Elghul

**Affiliations:** 1 Internal Medicine, Sheikh Shakhbout Medical City, Abu Dhabi, ARE

**Keywords:** aspiration, bronchial carcinoid, food bolus

## Abstract

Tracheobronchial aspiration is a very common and serious medical condition. It can present acutely with an aspiration pneumonia, or it can be chronic and occur over a long period of time. In some instances, the diagnosis can be missed, and patients may be treated for years for other medical conditions such as asthma, with no significant improvement. We present here a very interesting case of a 69-year-old gentleman with multiple comorbidities who presented with a fever and shortness of breath. He was initially diagnosed with aspiration pneumonia, but when he did not improve, a bronchoscopy was performed, which showed a mass in the right bronchus suspicious for a carcinoid tumor. However, a biopsy was taken and sent to pathology for analysis, which showed food material. He underwent a rigid bronchoscopy for mass removal, which indeed confirmed that the whole mass was composed of food material as a result of tracheobronchial aspiration.

## Introduction

A rare occurrence but a serious medical condition, tracheobronchial aspiration is when solids or liquids become retained in the airways of the lungs [1]. The clinical presentation of foreign body aspiration can range from chronic insidious lung damage to acute asphyxiation [2]. Common symptoms of aspiration include chronic cough, shortness of breath, and hemoptysis. A history of choking or regurgitation after feeding is a strong pointer towards aspiration; however, not all patients may have these clinical features [3,4]. As in our case presentation, there is often no clear history of aspiration, and diagnosis may become a challenge [5]. Several conditions and risk factors increase a patient’s risk for aspiration, and these include neurological dysfunction such as seizures, stroke, and Parkinson’s disease, alcoholism, facial trauma, and anatomical abnormalities of the pharynx and esophagus [6]. Often, patients with chronic aspiration are misdiagnosed with other conditions such as obstructive airway disease and do not respond satisfactorily to therapy [7]. In this report, we describe the case of a patient with a foreign body in the right bronchus misdiagnosed initially as pneumonia and later as lung malignancy.

## Case presentation

A 70-year-old gentleman who came for a visit from Sri Lanka to UAE in January 2020 presented in the emergency room on February 22, 2020, with complaints of subjective fever and cough productive of whitish sputum for three days prior to presentation. His symptoms were associated with generalized weakness, fatigue, and insomnia. He reported a history of an unintentional loss of 3 kg of weight secondary to decreased appetite over the past month. There were no symptoms of nausea, vomiting, shortness of breath, dysuria, or abdominal pain. There was no report of difficulty swallowing or coughing or regurgitation with food.

He was a poorly controlled type 2 diabetic with chronic kidney disease stage IIIA3 secondary to diabetic nephropathy and benign prostatic hyperplasia. His last hospitalization was about three months earlier, where he was admitted for investigation of right upper quadrant pain and was found to have gallstone disease. His surgical history is inclusive of a transurethral resection of the prostate about a year earlier, which failed, and he has been on indwelling urinary catheter ever since. His medications are atorvastatin, aspirin, tamsulosin, and gliclazide. He has no known allergies. He has a history of smoking five cigarettes a day for 40 years, but he quit smoking three years ago. The patient denied alcohol consumption or illicit drug use.

On physical examination, he seemed to be uncomfortable and tachypneic with a respiratory rate of 19 breaths per minute. His temperature was 38.2 degrees Celsius, pulse was 104 beats per minute, blood pressure was 152/65 mmHg, and oxygen saturation was 98% on 2 L of oxygen through nasal cannula. He was alert and oriented to time, place, and person, though he seemed to be in mild respiratory distress. He had no signs of anemia or jaundice. Neck examination showed no evidence of jugular venous distention. Chest examination revealed normal heart sounds without any murmurs. Auscultation showed reduced breath sounds over the right lower zone with minimal crepitations and dullness to percussion. The abdomen was soft and non-tender without any distention or organomegaly. Neurological examination elicited no focal defects.

**Figure 1:**
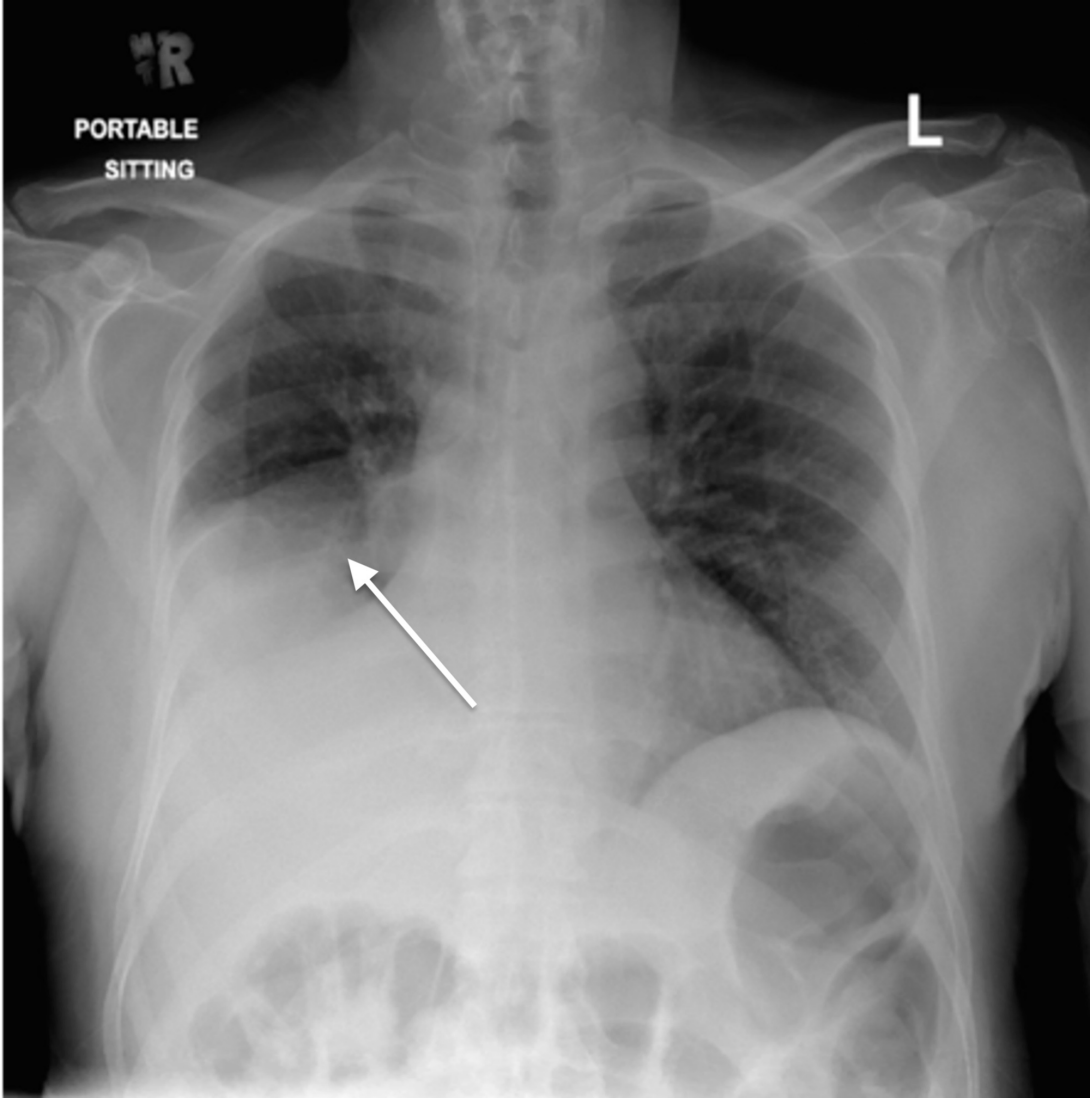
Chest X-ray on presentation

His labs showed lymphocytosis with a neutrophilic shift and elevation in inflammatory markers including C-reactive protein of 350 mg/L and procalcitonin of 27 ng/mL. His chest X-ray (Figure 1) showed opacification of the right lower lobe with elevation of the hemidiaphragm. Urine analysis was positive for leukocyte esterase and nitrites. He was admitted for the evaluation and management of urosepsis and healthcare-associated pneumonia, and he was started on piperacillin-tazobactam 2,000 mg IV every 6 hours and azithromycin 500 mg IV every 6 hours. His urine culture came back three days later growing pan-resistant *Pseudomonas aeruginosa* sensitive only to Colistin; however, that was not started due to his acute renal impairment. Furthermore, his urine was not the likely source of infection since he had been on an indwelling urinary catheter for a long period of time and is therefore expected to be colonized with multiple organisms.

Despite being on appropriate treatment for his presumed diagnosis of pneumonia, his respiratory status did not seem to be improving with persistent fever and rising inflammatory markers; his sputum and blood cultures revealed no growth. It was therefore decided to pursue CT of the chest with intravenous contrast for further evaluation of possible malignancy. This was not an easy decision considering his renal impairment, and it was discussed in detail with the patient and his family.

**Figure 2:**
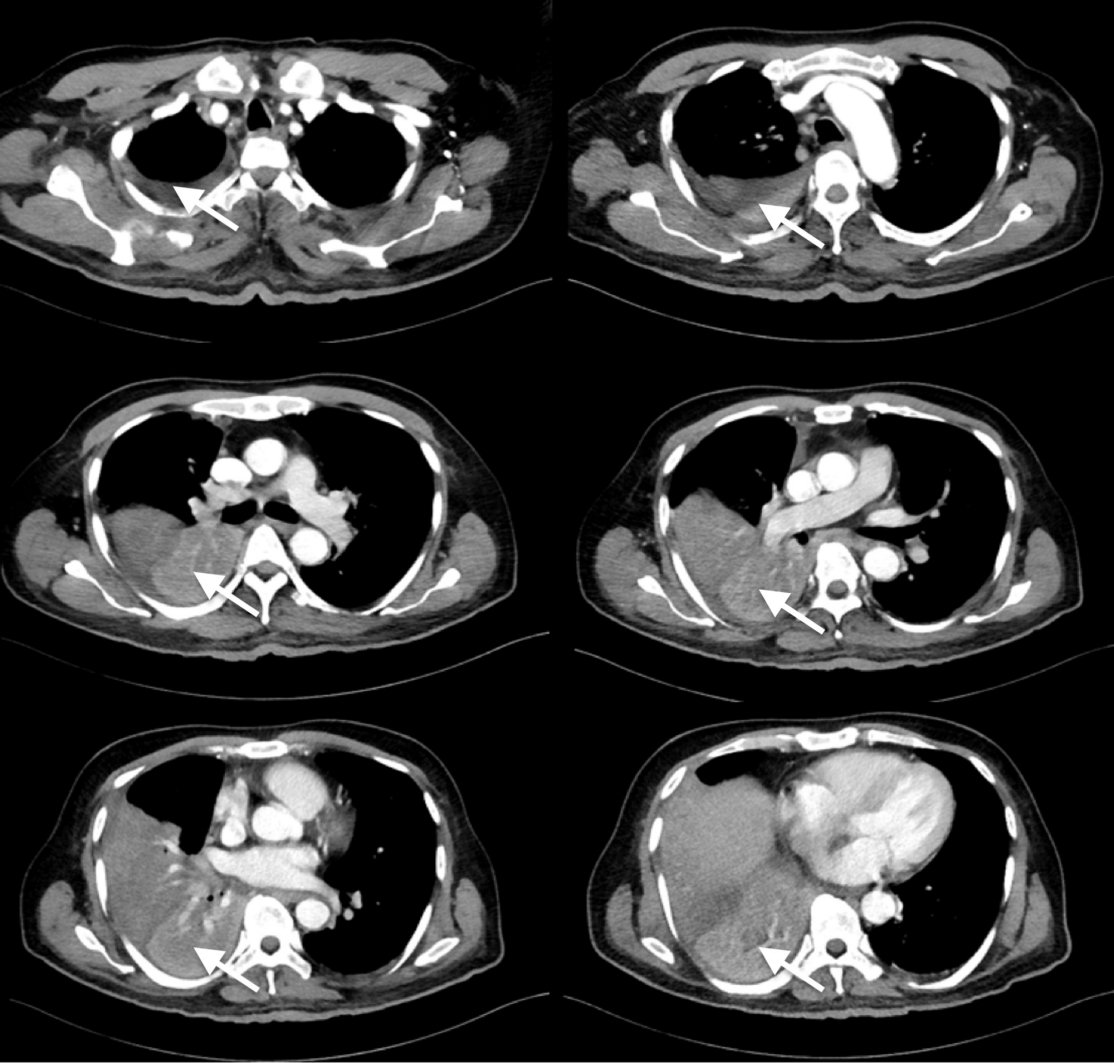
CT of the chest showing homogenously enhancing mass in the right bronchus intermedius causing collapse of the right lower lobe in addition to a small right-sided pleural effusion

CT of the chest (Figure 2) revealed a 1.4 cm x 1 cm homogenously enhancing mass in the right bronchus intermedius causing collapse of the right lower lobe in addition to a small right-sided pleural effusion. There was no report of lymphadenopathy or other focal nodules. The findings were suspicious for bronchial carcinoid. On further questioning, the patient reported no symptoms of flushing, diaphoresis, tremors, or diarrhea. Later on, urine 5-HIAA (5-hydroxyindoleacetic acid) test was performed, which came back normal.

**Figure 3:**
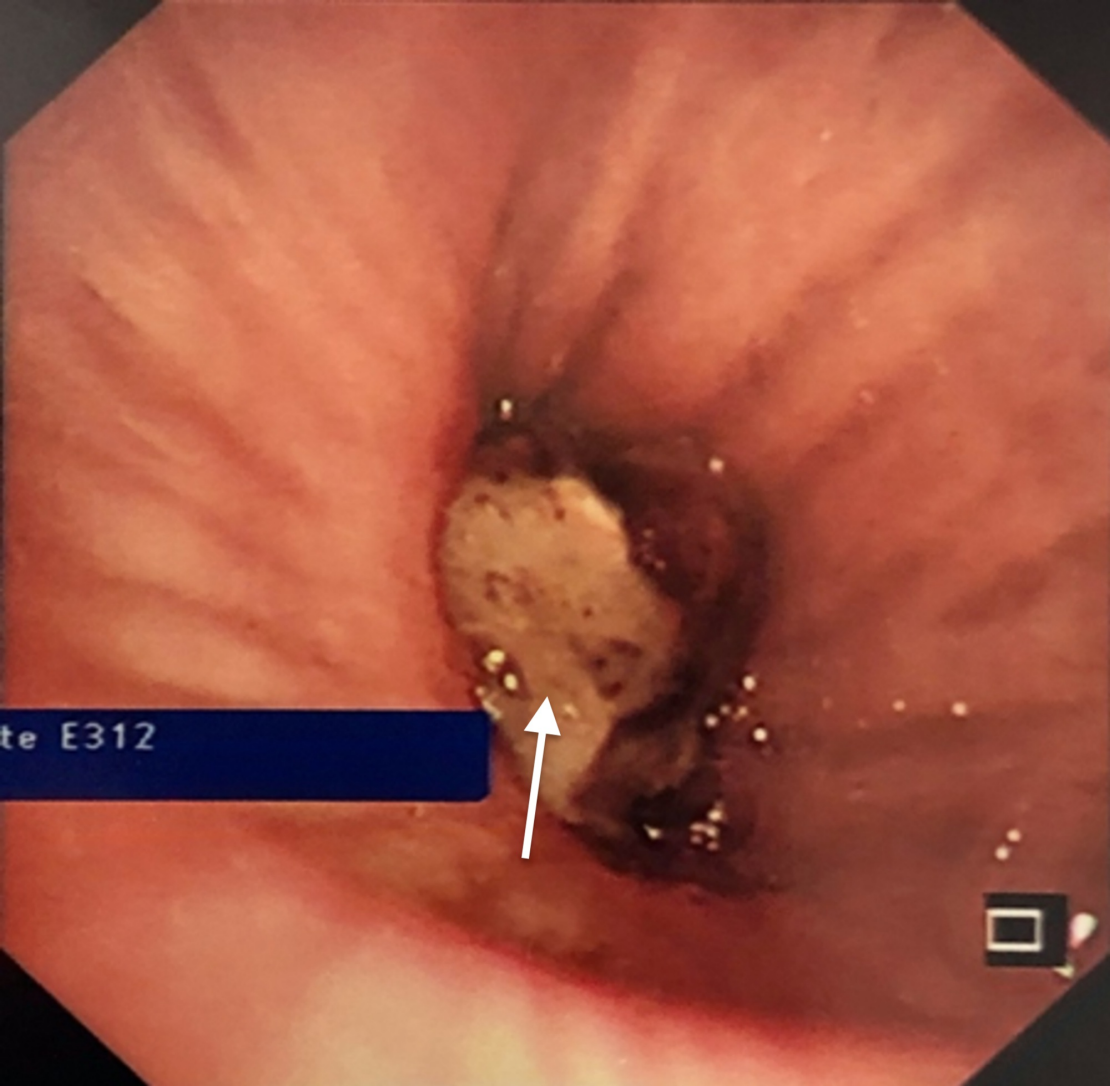
Mass seen in the right bronchus during bronchoscopy

After discussion with the pulmonologist, the patient was sent for evaluation with bronchoscopy and biopsy of the lung mass. The procedure confirmed the presence of a mass blocking the bronchus intermedius (Figure 3). The biopsy was reported two days later as impacted food material. His antibiotics were discontinued at this point, and he was referred to swallowing and vocal cord evaluation, which showed intermittent disorganized lingual movements and a high aspiration risk. It was recommended that he starts a dysphagia diet with thickened fluids and pureed solid materials.

**Figure 4:**
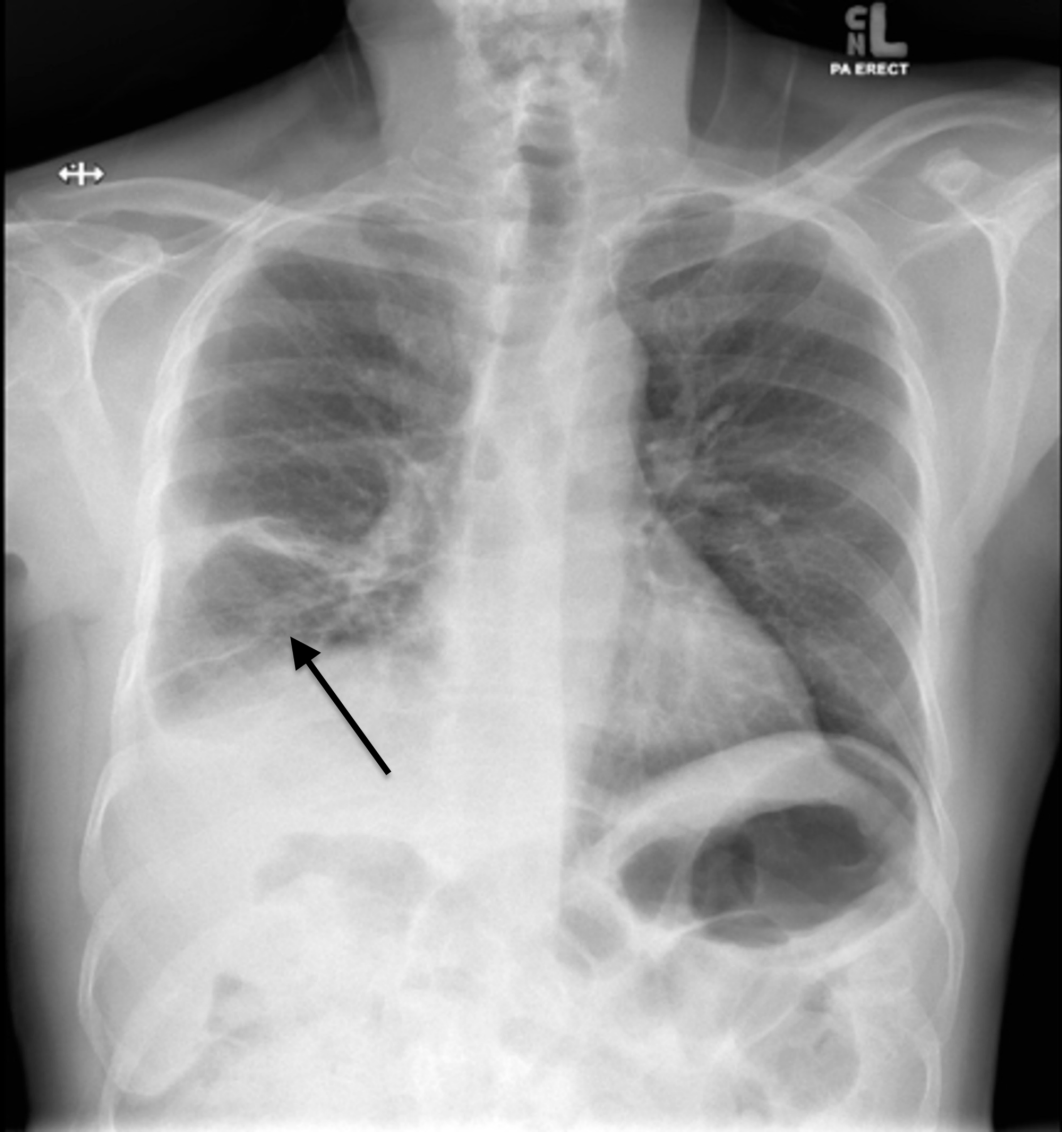
Chest X-ray on follow-up after foreign body removal

The patient later underwent rigid bronchoscopy for the retrieval of foreign material by thoracic surgery. After removal, the patient’s white cell count and inflammatory markers started trending down, and he started feeling much better. During his follow-up two months later, a repeat chest X-ray was performed, which showed improvement and re-expansion of the collapsed lung (Figure 4).

## Discussion

In healthy adults, foreign body aspiration is uncommon, whereas it more commonly occurs in children and the elderly [3]. Adults who develop foreign body aspiration often have one of several conditions and risk factors, which increase a patient’s risk for aspiration, and these include neurological dysfunction such as seizures, stroke, and Parkinson’s disease, alcoholism, facial trauma, and anatomical abnormalities of the pharynx and esophagus [6]. The swallowing and cough reflexes are essential defense mechanisms, which protect from aspiration; however, if they get bypassed or suppressed, patients develop an increased aspiration risk [3,4,7]. Aspirated foreign bodies may remain in the main bronchi or their branches, leading to obstruction, or they may even reach the lung. Due to its more vertical path, the right main bronchus is the most common culprit [1].

The clinical presentation of foreign body aspiration can vary widely from an indolent chronic cough in up to two-thirds of patients to more life-threatening emergencies including upper airway obstruction with respiratory failure requiring urgent intervention [1]. Patients may also present with intermittent choking sensation associated with a dry cough, which indeed raises the suspicion for aspiration; however, the majority of patients do not present like this [3]. Other clinical features include wheezing, hemoptysis, and shortness of breath [8]. Clinical outcomes can vary from immediate resolution to recurrent pulmonary disease or even death. Sometimes, as is the case in our patient, there are no features from history or clinical assessment to suggest aspiration, and further workup is required [5].

The most commonly aspirated foreign bodies include food particles, broken fragments of teeth, plastic objects, metallic objects including pins, screws, and needles [9]. A series of 3,217 cases over 26 years showed that in children, the most commonly aspirated objects were peanuts, vegetable matter, and toy parts. However, the series demonstrated that adults more commonly aspirated food particles, medication tablets, and dental pieces [10].

Foreign bodies that are radiologically opaque can be directly visualized on chest X-ray unless obscured by parenchymal changes [11]. Other radiological features of foreign body aspiration include atelectasis, unilateral hyperinflation, unresolving pneumonia, and localized bronchiectasis [11]. In addition, CT scans of the chest may reveal an intra-parenchymal or intra-bronchial mass, as was the case in our patient [8].

If the diagnosis is not clear after initial workup, the definitive diagnosis of foreign body aspiration can be confirmed when visualized directly using indirect laryngoscopy or bronchoscopy [12]. Furthermore, the foreign body can be retrieved using grasping forceps during fiberoptic or rigid bronchoscopy, as was the case in our patient [3,6]. However, in cases of chronic aspiration, the foreign body may not be visualized clearly on bronchoscopy, which would instead show features of tissue reaction to the foreign body including endobronchial stenosis, granulation tissue, strictures, and edema [1,12].

As in our patient, establishing the diagnosis of foreign body aspiration can pose a challenge due to multiple reasons [13]. A large proportion of patients may not give a clear history of aspiration or of any risk factors that would suggest aspiration. Furthermore, some patients may present months to years after the aspiration event [14]. Moreover, some patients may develop subtle clinical symptoms and their aspiration may remain undetected for many years [15].

In some patients, aspiration is not recognized and they are misdiagnosed as asthma, emphysema, chronic pneumonia, or malignancy, similar to what happened with our patient [8,16]. This should raise our suspicion for occult foreign body aspiration in cases of chronic or recurrent pneumonia, which do not respond to antibiotic therapy [16]. In other instances, foreign bodies may be incidentally discovered when bronchoscopy is performed for evaluation of a chronic cough, hemoptysis, or an unresolving pneumonia [17].

Removal of the foreign body is usually required for definitive treatment, and the first step in doing so is a flexible bronchoscopy [18]. However, this is successful initially in around 90% of patients, who will require further management with a rigid bronchoscopy, as was the case in our patient. Rigid bronchoscopy is usually pursued in cases where flexible bronchoscopy fails or is found inadequate for simultaneous safe extraction and airway management [18]. If bronchoscopy shows significant granulation tissue or airway stenosis and extraction is not possible, other modalities such as endobronchial ablation or cryotherapy may be attempted [19].

Delaying the diagnosis of foreign body aspiration, which results in inappropriate management, can lead to chronic complications including recurrent pneumonia, bronchiectasis, strictures, and development of inflammatory polyps at the site of obstruction [1].

## Conclusions

To conclude, foreign body aspiration is not always a clear and easy diagnosis to make and may pose a diagnostic challenge to the clinician. It should be on the list of differential diagnoses for patients with unexplained respiratory symptoms or those with pneumonias that are recurrent or unresolving. Our patient describes a case of an elderly gentleman who was diagnosed initially with an unresolving pneumonia and later with possible malignancy; however, the foreign body was found incidentally on bronchoscopy, and the diagnosis was confirmed pathologically. The patient improved significantly after the foreign body was removed.
